# Impact of renal dysfunction on immediate versus staged revascularization of non-culprit lesions in patients with ST segment elevation myocardial infarction: a pre-specified subgroup analysis of the randomized MULTISTARS AMI trial

**DOI:** 10.1007/s00392-026-02875-w

**Published:** 2026-03-09

**Authors:** Miklos Rohla, Kurt Huber, Ferdinando Varbella, Axel Linke, Bettina Schwarz, Stephan B. Felix, Moritz Seiffert, Rahel Kesterke, Peter Nordbeck, Bernhard Witzenbichler, Irene M. Lang, Mirjam Kessler, Christian Valina, Alban Dibra, Philipp Jakob, Marco Moccetti, Matteo Vercellino, Luise Gaede, Lorenz Bott-Flügel, Julia Stehli, Alessandro Candreva, Francesco Paneni, Christian Templin, Matthias Schindler, Manfred Wischnewsky, Greca Zanda, Giorgio Quadri, Norman Mangner, Aurel Toma, Giulia Magnani, Peter Clemmensen, Thomas F. Lüscher, Thomas Münzel, P. Christian Schulze, Karl-Ludwig Laugitz, Wolfgang Rottbauer, Franz-Josef Neumann, Steffen Schneider, Thomas Riemer, Franz Weidinger, Stephan Achenbach, Gert Richardt, Adnan Kastrati, Ian Ford, Frank Ruschitzka, Barbara E. Stähli

**Affiliations:** 1 Department of Cardiology, Bern University Hospital, University of Bern, Bern, Switzerland; 2 Austrian Heart Foundation, Vienna, Austria; 3 Medical Private University Burgenland (GmbH), Pinkafeld, Austria; 4 Interventional Cardiology Unit, Degli Infermi Hospital, Rivoli, Turin, Italy; 5 Department of Internal Medicine and Cardiology, Technische Universität Dresden, University Clinic, Herzzentrum Dresden, Dresden, Germany; 6 Heart Center, Segeberger Kliniken GmbH, Academic Teaching Hospital for the Universities of Kiel, Lübeck and Hamburg, Bad Segeberg, Germany; 7 Department of Internal Medicine B, DZHK (German Centre for Cardiovascular Research), University Medicine Greifswald, Partner Site Greifswald, Greifswald, Germany; 8 BG University Hospital Bergmannsheil, Ruhr-University Bochum, Bochum, Germany; 9 Department of Cardiology, Center for Translational and Experimental Cardiology (CTEC), University Heart Center, University Hospital Zurich, University of Zurich, Zurich, Switzerland; 10 Department of Internal Medicine I, University Hospital Würzburg, Würzburg, Germany; 11 Department of Cardiology and Pneumology, Helios Amper-Klinikum, Dachau, Germany; 12 Division of Cardiology, Department of Internal Medicine II, Medical University of Vienna, Vienna, Austria; 13 Department of Cardiology, Ulm University Heart Center, Ulm, Germany; 14 Department of Cardiology and Angiology, Medical Center, University of Freiburg, Freiburg, Germany; 15 Faculty of Medicine, University of Freiburg, Freiburg, Germany; 16 Department of Cardiovascular Diseases, University of Medicine, Tirana University Hospital “Mother Teresa”, Tirana, Albania; 17 Cardiocentro Ticino Institute, Ente Ospedaliero Cantonale, Lugano, Switzerland; 18 Cardiovascular Disease and Cardiology Unit, IRCCS, Policlinico San Martino University Hospital, Genoa, Italy; 19 Klinikum Herrsching, Starnberger-Kliniken, Herrsching, Germany; 20 Cardiology and Pneumology, Klinikum Landkreis Erding, Erding, Germany; 21 FB Mathematics and Computer Science, University of Bremen, Bremen, Germany; 22 Division of Cardiology, Parma University Hospital, Parma, Italy; 23 Department of Cardiology, Center for Population Health Innovation (POINT), University Heart and Vascular Center HamburgUniversity Clinic Hamburg-Eppendorf (UKE), andDZHK (German Centre for Cardiovascular Research) Partner Site Hamburg/Kiel/Lübeck, Hamburg, Germany; 24 Heart Division, Royal Brompton &, Harefield Hospitals and National Heart and Lung Institute, Imperial College London, London, UK; 25 Department of Cardiology, Center for Cardiovascular Research, University Medical Center Mainz, Johannes Gutenberg University, Partner Site Rhine Main, Mainz, Germany; 26 Department of Internal Medicine I, Division of Cardiology, Pneumology, Angiology and Intensive Medical Care, University Hospital Jena, Friedrich-Schiller-University Jena, Jena, Germany; 27 Clinic and Policlinic Internal Medicine I (Cardiology and Angiology), DZHK (German Center for Cardiovascular Research), Technische Universität Münchenand, Partner Site Munich Heart Alliance, Klinikum Rechts Der Isar, Munich, Germany; 28 Institut Für Herzinfarktforschung (Foundation IHF), Ludwigshafen, Germany; 29 Medical Department With Cardiology and Intensive Care Medicine, Hospital Landstrasse, Vienna, Austria; 30 Klinik Für Herz Und Kreislauferkrankungen, DZHK (German Center for Cardiovascular Research), Deutsches Herzzentrum München, Technische Universität Münchenand, Partner Site Munich Heart Alliance , Munich, Germany; 31 Robertson Centre for Biostatistics, University of Glasgow, Glasgow, UK

**Keywords:** Acute coronary syndrome myocardial infarction, Percutaneous coronary intervention, Chronic kidney disease, Acute kidney injury, Contrast-induced nephropathy, Renal failure

## Abstract

**Background:**

Renal dysfunction might affect outcomes in patients with ST-segment elevation myocardial infarction (STEMI) and multivessel coronary artery disease (MVD) undergoing percutaneous coronary intervention (PCI).

**Methods:**

In MULTISTARS AMI, patients with STEMI and MVD were randomized to immediate or staged PCI of non-culprit lesions. In this pre-specified analysis, patients were stratified according to the presence of renal dysfunction at baseline, defined at an estimated glomerular filtration rate (eGFR) of 60 ml/min/1.73 m^2^. Patients with an eGFR < 30 ml/min/1.73 m^2^ were excluded from the trial. The primary endpoint was a composite of death, non-fatal myocardial infarction, stroke, unplanned revascularization, or hospitalization for heart failure at 1 year.

**Results:**

In MULTISTARS AMI, 108 (13%) of 832 patients had renal dysfunction. The primary endpoint occurred more frequently in patients with renal dysfunction (19.4% vs. 11.2%, unadjusted HR 1.82, 95% CI 1.13–2.94), primarily driven by higher rates of death. Among patients with renal dysfunction, the rates of the primary end point were 14.5% and 24.5% in the immediate and staged PCI groups (unadjusted HR 0.55, 95% CI 0.23–1.33). There was no interaction between renal dysfunction and the randomized treatment assignment with respect to the primary end point (adjusted HR 1.30, 95% CI 0.8–2.20, p_int_ 0.82). The occurrence of acute renal insufficiency was statistically similar in patients with renal dysfunction who underwent immediate and staged PCI (10.9% vs. 18.9%, unadjusted HR 0.61, 95% CI 0.22–1.72, p_int_ 0.09). Renal dysfunction at baseline emerged as a strong risk factor for the development of acute renal insufficiency (adjusted HR 5.0, 95% CI 2.30–10.70, *p* < 0.01).

**Conclusions:**

Outcomes with immediate compared to staged multivessel PCI did not appear significantly altered by the presence of renal dysfunction at baseline. (Supported by Boston Scientific; MULTISTARS AMI ClinicalTrials.gov number, NCT03135275).

**Graphical Abstract:**

**Figure:**
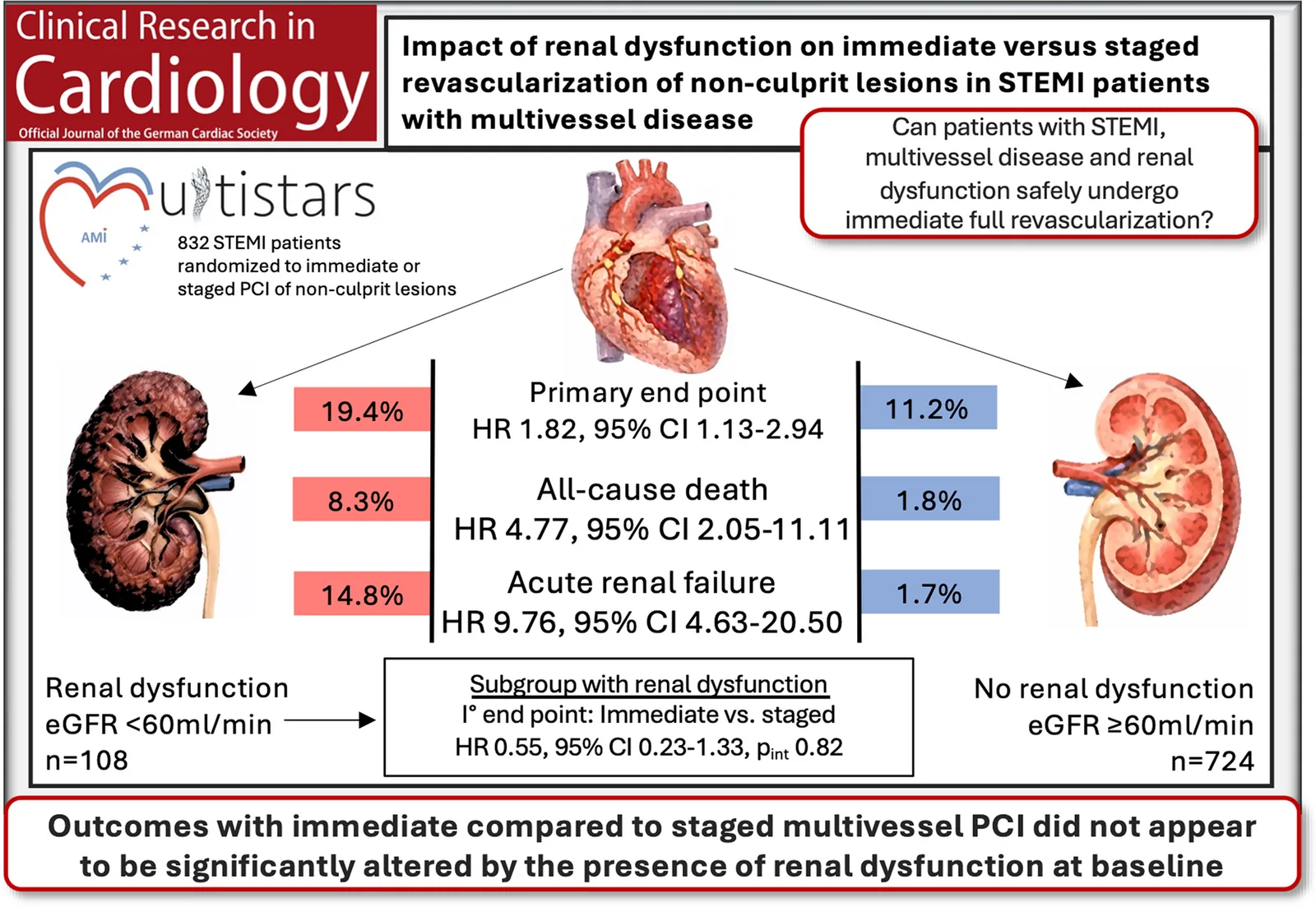

**Supplementary Information:**

The online version contains supplementary material available at https://doi.org/10.1007/s00392-026-02875-w.

## Introduction

Chronic kidney disease (CKD) is associated with higher rates of ischemic events, an increased risk of bleeding, and subsequently worse overall prognosis in patients with acute coronary syndromes (ACS) [1, 2]. In addition to the elevated cardiovascular risk profile of patients with renal dysfunction, contrast-induced nephropathy (CIN) after percutaneous coronary intervention (PCI) using iodinated contrast agents warrants further consideration [3]. Tubular toxicity, microvascular alterations, oxidative stress, inflammation, and protein precipitation have been described as potentially causal mechanisms [1, 2]. While the incidence of acute kidney injury (AKI) in acute myocardial (AMI) infarction cohorts is relatively low (< 5%), the amount of contrast volume used appears to be associated with the development of CIN, and patients who develop AKI or CIN after PCI have substantially worse short- and long-term outcomes, including a higher risk of death [2, 4–8].

The 2023 European Society of Cardiology Guidelines on the Management of Patients with ACS recommended to apply the same diagnostic and therapeutic strategies irrespective of CKD. This recommendation is largely based on expert opinion, and there is limited evidence with respect to non-culprit lesion PCI strategies in patients with CKD [9].

This pre-specified subgroup analysis of the Multivessel Immediate versus Staged Revascularization in Acute Myocardial Infarction (MULTISTARS AMI) trial is addressing the safety and efficacy of an immediate as compared to a staged PCI strategy for non-culprit lesions in patients with and without renal dysfunction at baseline.

## Methods

The design and methods of the MULTISTARS AMI trial have been previously described [10, 11]. Briefly, MULTISTARS AMI was an investigator-initiated, multinational, randomized, open-label trial that evaluated a strategy of immediate multivessel PCI as compared with a strategy of staged multivessel PCI in hemodynamically stable patients with ST segment elevation myocardial infarction (STEMI) and multivessel coronary artery disease (MVD), who had undergone successful primary PCI of the infarct-related coronary artery. From October 2016 through June 2022, a total of 840 patients with STEMI were enrolled in the study at 37 sites in Europe and randomly assigned in a 1:1 ratio to either immediate or staged multivessel PCI. In the immediate group, PCI of non-culprit lesions was performed immediately after revascularization of the infarct-related artery during the same procedure. In the staged group, PCI of non-culprit lesions was performed within 19 to 45 days after revascularization of the infarct-related artery. A third-generation, biodegradable-polymer, everolimus-eluting synergy stent (Boston Scientific Corporation, Marlborough, MA) was recommended for PCI. Main exclusion criteria consisted of stent thrombosis, in-stent restenosis, left main stenosis ≥ 50% or left main equivalent, chronic total occlusion of a major coronary artery, and need for emergency coronary artery bypass grafting (CABG), previous CABG, and severe renal failure (eGFR < 30 mL/min) or dialysis. Follow-up was performed at 30 days, 6 months, and 1 year.

For the purpose of this pre-specified sub-group analysis and in accordance with the Kidney Disease Improving Global Outcomes (KDIGO) classification, patients were stratified according to the presence of renal dysfunction at an estimated glomerular filtration rate (eGFR) threshold of 60 ml/min/1.73 m^2^, using the first available blood draw performed during the index hospitalization [12]. Estimated glomerular filtration rate was calculated using the Chronic Kidney Disease Epidemiology Collaboration (CKD-EPI) equation [13].

The study was approved by an institutional review board in each country and carried out in accordance with national law and the Declaration of Helsinki of 1975 (in its current revised form). All patients provided written informed consent.

### End points

The primary end point was a composite of all-cause death, non-fatal myocardial infarction (MI), stroke, unplanned ischemia-driven revascularization, or hospitalization for heart failure within 1 year after randomization. Secondary end points included individual components of the primary end point, death or non-fatal myocardial infarction, cardiac death or non-fatal MI, target vessel revascularization, target lesion revascularization, stent thrombosis, acute renal insufficiency or renal replacement therapy, major bleeding (Bleeding Academic Research Consortium [BARC] type 3 or 5), procedural success, and quality of life (QoL).

Acute renal insufficiency was defined as ≥ 0.3 mg/dl or ≥ 50% increase in creatinine levels from baseline in accordance with the KDIGO classification [13]. Renal replacement therapy was defined as any use of dialysis, hemofiltration or hemodiafiltration for volume overload, hyperkalemia, uremia, or metabolic acidosis.

### Statistical analysis

Binary, categorical, and ordinal variables are described using absolute numbers and percentages, and continuous variables by their medians and quartiles. Differences between groups were assessed using the Pearson’s chi^2^ test or Wilcoxon’s test, respectively. Time-to-event end points were analyzed considering always the first event per subject, if any. Event-free subjects were censored at their last known contact date. Cumulative incidence function (CIF) was calculated to visualize event rates during a 1-year follow-up period. Outcomes not including death were adjusted for the competing risk of death. Gray’s test was applied to assess differences of CIF between subgroups. Effect sizes are presented as hazard ratios (HR, for time-to-event end points) or odds ratios (OR, for procedural success) with corresponding 95% confidence intervals (CI). The Wilcoxon–Mann–Whitney (WMW) variant of OR was applied for quality of life (QoL) data. Confidence intervals were calculated using the methods of Fine and Gray, Wald, or WMW, respectively. Adjustment for confounders was performed using a Cox regression model with stepwise selection of pre-specified variables. *P* values of 0.2 were required for each predictor to enter the model and 0.25 to stay in it. Renal dysfunction, age, BMI, sex, hypertension, diabetes, and dyslipidemia were forced into each model, whenever a sufficient number of events allowed numerically stable estimates. The only imputation of data was performed in the case of missing QoL data. The EQ-5D-5L index of dead subjects was substituted by the minimum possible value of zero. Natural cubic splines over eGFR were performed as adjunct sensitivity analysis for continuous representation of renal dysfunction. All shown *p* values should be interpreted descriptively. All statistical analyses were performed with the use of SAS software, version 9.4, for Windows (SAS Institute).

## Results

### Patient characteristics

Baseline characteristics are presented in Table 1. Of 840 patients who were enrolled in the MULTISTARS AMI trial, baseline eGFR data at index hospitalization were available in 832 patients (99%). Estimated glomerular filtration rate was 49.4 (41.2–55.5) ml/min/1.73 m^2^ in patients with renal dysfunction an 86.8 (76.3–95.8) ml/min/1.73 m^2^ in those without.

**Table 1:** Baseline characteristics

Characteristics	Renal dysfunction (eGFR < 60 ml/min) *N* = 108	No renal dysfunction (eGFR ≥ 60 ml/min) *N* = 724	*p* value
Number of patients	108 (13.0%)	724 (87.0%)	
Age—years	76.5 (68.5, 81.0)	64.0 (56.0, 71.0)	<.01
Male sex	68/108 (63.0%)	588/724 (81.2%)	<.01
Race/ethnicity			0.71^X2^
Caucasian	107/108 (99.1%)	710/724 (98.1%)	
Black	0/108 (0.0%)	3/724 (0.4%)	
Asian	1/108 (0.9%)	11/724 (1.5%)	
eGFR (CKD-EPI) [ml/min/1.73m^2^]	49.4 (41.2, 55.5)	86.8 (76.3, 95.8)	<.01
Hypertension	75/108 (69.4%)	362/724 (50.0%)	<.01
Diabetes	31/108 (28.7%)	98/724 (13.5%)	<.01
Dyslipidemia	39/108 (36.1%)	184/722 (25.5%)	0.02
Family history of CAD	16/107 (15.0%)	204/722 (28.3%)	<.01
Smoking history	36/106 (34.0%)	386/722 (53.5%)	<.01
Former	18/106 (17.0%)	117/720 (16.3%)	
Current	18/106 (17.0%)	267/720 (37.1%)	
Previous PCI	9/108 (8.3%)	47/723 (6.5%)	0.48
Previous myocardial infarction	8/108 (7.4%)	40/722 (5.5%)	0.44
Previous stroke	5/108 (4.6%)	13/724 (1.8%)	0.06
Peripheral artery disease	6/108 (5.6%)	13/723 (1.8%)	0.01
Resuscitation prior to hospital arrival	3/108 (2.8%)	28/724 (3.9%)	0.58
Left bundle branch block	2/106 (1.9%)	8/711 (1.1%)	0.51
Location of myocardial infarction			
Anterior	39/106 (36.8%)	284/701 (40.5%)	0.47
Inferior	48/106 (45.3%)	292/701 (41.7%)	0.48
Lateral	9/106 (8.5%)	91/701 (13.0%)	0.19
Posterior	26/106 (24.5%)	145/701 (20.7%)	0.37

Values are median (Q1––Q3), *n* (%), or *n*/*N* (%)

*CAD*, coronary artery disease; *CKD-EPI*, chronic kidney disease epidemiology collaboration; *eGFR*, estimated glomerular filtration rate; *PCI*, percutaneous coronary intervention

Patients with renal dysfunction were older, more likely female, more often suffered from cardiovascular risk factors such as hypertension, diabetes, or dyslipidemia, and more frequently presented with peripheral artery disease. Oral anticoagulants, antidiabetics, and insulin were more frequently prescribed in patients with renal dysfunction. The use of secondary prevention therapies including statins, beta-blockers, and angiotensin converting enzyme (ACE) inhibitors or angiotensin-receptor blockers were comparable between groups (Supplementary Table 1).

Procedural characteristics were comparable between groups, with respect to the number of vessels with relevant non-culprit lesions, number of stents used, total stent length, vascular access site, and use of functional or intracoronary imaging guidance (Supplementary Table 2). The amount of contrast volume used was similar between groups for the index procedure, but overall lower in patients with renal dysfunction when including both the index and staged procedures (Supplementary Table 2).

### Primary and secondary end points in patients with and without renal dysfunction

**Fig. 1:**
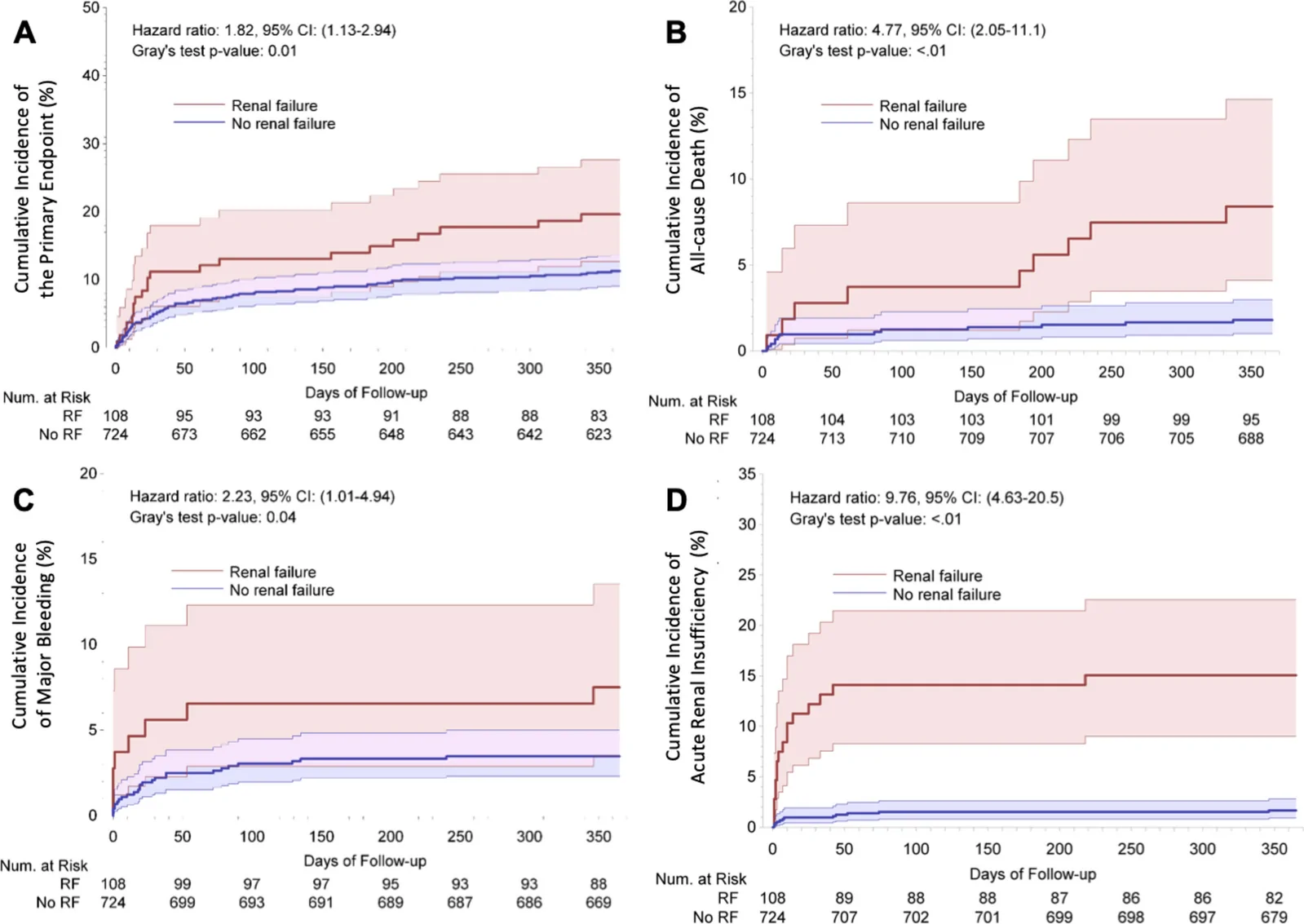
Primary and secondary end points in patients with and without renal dysfunction at baseline. **A** Primary composite endpoint of death, non-fatal myocardial infarction, stroke, unplanned ischemia-driven revascularization, or hospitalization for heart failure. **B** All-cause death. **C** Major bleeding, defined as Bleeding Academic Research Consortium grade 3 or 5. **D** Acute renal insufficiency or renal replacement therapy

The distribution of patients with (*n* = 108) and without renal dysfunction (*n* = 724), who were randomized to immediate versus staged multivessel PCI was balanced (Table 3). The primary end point, a composite of death, non-fatal MI stroke, unplanned ischemia-driven revascularization, or hospitalization for heart failure at 1 year occurred more frequently in patients with as compared to those without renal dysfunction (19.4% vs. 11.2%, unadjusted HR 1.82, 95% CI 1.13–2.94, Fig. 1). This was primarily driven by higher rates of all-cause death (8.3% vs. 1.8%, unadjusted HR 4.77, 95% CI 2.05–11.11, Fig. 1), with a trend towards increased rates of non-cardiovascular death (4.6% vs. 0.8%, unadjusted HR 5.73, 95% CI 1.76–18.70) as opposed to cardiac death (2.8% vs. 1.0%, unadjusted HR 2.91, 95% CI 0.76–11.18). Rates of major bleeding (Bleeding Academic Research Consortium [BARC] type 3 or 5) were higher in patients with renal dysfunction (7.4% vs. 3.5%, unadjusted HR 2.23, 95% CI 1.01–4.94, Fig. 1). The incidence of acute renal insufficiency or renal replacement therapy was more than ninefold higher in patients with as compared to those without renal dysfunction at baseline (14.8% vs 1.7%, unadjusted HR 9.76, 95% CI 4.63–20.50, Fig. 1). Other secondary end points are presented in Table 2.

**Table 2:** Primary and secondary end points, stratified by the presence of renal dysfunction

	**Renal dysfunction** **(eGFR < 60 ml/min)**	**No renal dysfunction** **(eGFR ≥ 60 ml/min)**	Unadjusted effect of renal dysfunction*
**Primary end point**^**1**^			
Death, non-fatal myocardial infarction, stroke, unplanned ischemia-driven revascularization, or hospitalization for heart failure	21/108 (19.4%)	81/724 (11.2%)	1.82 (1.13–2.94)
**Secondary end points at 1 year**^**1**^			
Death	9/108 (8.3%)	13/724 (1.8%)	4.77 (2.05–11.11)
Non-fatal myocardial infarction	3/108 (2.8%)	27/724 (3.7%)	0.76 (0.23–2.54)
Stroke	2/108 (1.9%)	10/724 (1.4%)	1.36 (0.30–6.17)
Unplanned ischemia-driven revascularization	8/108 (7.4%)	48/724 (6.6%)	1.15 (0.54–2.44)
Hospitalization for heart failure	3/108 (2.8%)	8/724 (1.1%)	2.57 (0.69–9.62)
Death or non-fatal myocardial infarction	12/108 (11.1%)	38/724 (5.2%)	2.19 (1.15–4.19)
Cardiac death	3/108 (2.8%)	7/724 (1.0%)	2.91 (0.76–11.18)
Non-cardiovascular death	5/108 (4.6%)	6/724 (0.8%)	5.73 (1.76–18.70)
Cardiac death or non-fatal myocardial infarction	6/108 (5.6%)	32/724 (4.4%)	1.30 (0.54–3.13)
Target vessel revascularization	3/108 (2.8%)	19/724 (2.6%)	1.08 (0.32–3.66)
Target lesion revascularization	3/108 (2.8%)	18/724 (2.5%)	1.14 (0.34–3.84)
Stent thrombosis	3/108 (2.8%)	7/724 (1.0%)	2.92 (0.76–11.24)
Acute renal insufficiency or renal replacement therapy	16/108 (14.8%)	12/724 (1.7%)	9.76 (4.63–20.50)
Major bleeding (BARC type 3 or 5)	8/108 (7.4%)	25/724 (3.5%)	2.23 (1.01–4.94)
Procedural success^**2**^	78/89 (87.6%)	572/627 (91.2%)	0.68 (0.34–1.36)
Quality of life			
EQ-5D-5L index^**3**^	0.832 ± 0.314, *N* = 101	0.925 ± 0.178, *N* = 698	0.75 (0.59–0.95)

*BARC*, bleeding academic research consortium; *EQ-5D-5L*, European Quality of life-5 dimension-5 level; *eGFR*, estimated glomerular filtration rate

^*^Unadjusted effects are described by hazard ratios or odds ratios and their 95% confidence intervals

^1^Hazard ratio and 95% confidence interval, Fine and Gray method, considering competing risk all-cause death

^2^Odds ratio and 95% confidence interval, Wald method

^3^Wilcoxon–Mann–Whitney odds ratio and 95% confidence interval

### Primary end point in patients undergoing immediate or staged multivessel PCI

**Fig. 2:**
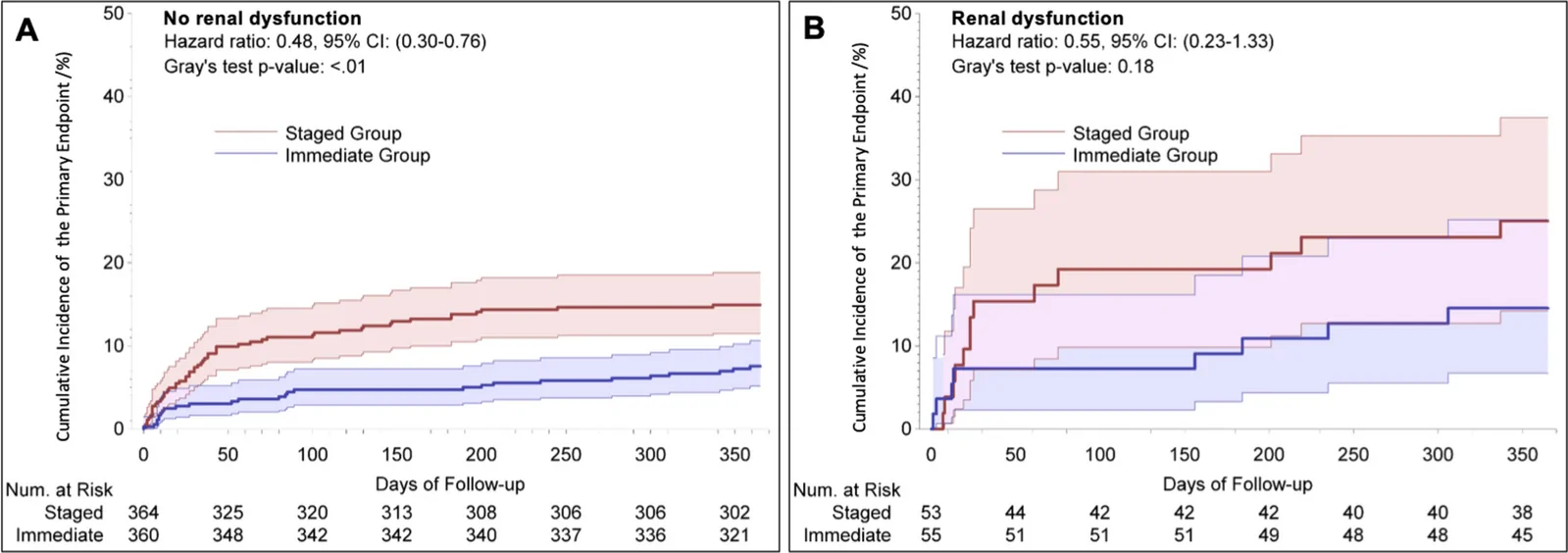
Primary end points in patients undergoing immediate or staged revascularization of non-culprit lesions, stratified by renal dysfunction at baseline. **A** Subgroup without renal dysfunction. **B** Subgroup with renal dysfunction

Immediate multivessel PCI was non-inferior to staged multivessel PCI in patients with (unadjusted HR 0.55, 95% CI 0.23–1.33) and in those without renal dysfunction (unadjusted HR 0.48, 95% CI 0.30–0.76, Table 3**, **Fig. 2).

**Table 3:** Primary and secondary end points in patients undergoing immediate or staged revascularization, stratified by the presence of renal dysfunction

	**Renal dysfunction (eGFR < 60 ml/min)** ***n* = 108**			**No renal dysfunction (eGFR ≥ 60 ml/min)** ***n* = 724**				
	**Immediate** ***n* = 55**	**Staged** ***n* = 53**	Unadjusted main effects*	**Immediate** ***n* = 360**	**Staged** ***n* = 364**	Unadjusted main effects*	**Adjusted HRs (95% CIs)**^**†**^	***p* for interaction**
**Primary end point**^**1**^								
Death, non-fatal myocardial infarction, stroke, unplanned ischemia-driven revascularization, or hospitalization for heart failure	8/55 (14.5%)	13/53 (24.5%)	0.55 (0.23–1.33)	27/360 (7.5%)	54/364 (14.8%)	0.48 (0.30–0.76)	1.3 (0.8–2.2)	0.82
**Secondary end points at 1 year**^**1**^								
Death	5/55 (9.1%)	4/53 (7.5%)	1.17 (0.32–4.33)	7/360 (1.9%)	6/364 (1.6%)	1.18 (0.40–3.50)	1.7 (0.7–4.1)	0.99
Non-fatal myocardial infarction	2/55 (3.6%)	1/53 (1.9%)	1.88 (0.17–20.39)	6/360 (1.7%)	21/364 (5.8%)	0.28 (0.11–0.69)	0.6 (0.2–2.6)	0.13
Stroke	1/55 (1.8%)	1/53 (1.9%)	0.94 (0.06–14.53)	4/360 (1.1%)	6/364 (1.6%)	0.67 (0.19–2.37)	0.5 (0.1–2.3)	0.81
Unplanned ischemia-driven revascularization	3/55 (5.5%)	5/53 (9.4%)	0.54 (0.13–2.25)	14/360 (3.9%)	34/364 (9.3%)	0.40 (0.21–0.74)v	1.2 (0.5–2.8)	0.67
Hospitalization for heart failure	0/55 (0.0%)	3/53 (5.7%)	-	5/360 (1.4%)	3/364 (0.8%)	1.68 (0.40–7.02)	1.0 (0.3–3.7)	< 0.01
Death or non-fatal myocardial infarction	7/55 (12.7%)	5/53 (9.4%)	1.34 (0.43–4.19)	12/360 (3.3%)	26/364 (7.1%)	0.45 (0.23–0.90)	1.2 (0.6–2.5)	0.11
Cardiac death	1/55 (1.8%)	2/53 (3.8%)	0.48 (0.04–5.28)	4/360 (1.1%)	3/364 (0.8%)	1.34 (0.30–5.99)	1.8 (0.4–7.8)	0.53
Non-cardiovascular death	3/55 (5.5%)	2/53 (3.8%)	1.38 (0.23–8.16)	3/360 (0.8%)	3/364 (0.8%)	1.01 (0.20–4.99)	1.4 (0.4–5.2)	0.96
Cardiac death or non-fatal myocardial infarction	3/55 (5.5%)	3/53 (5.7%)	0.97 (0.20–4.77)	9/360 (2.5%)	23/364 (6.3%)	0.38 (0.18–0.83)	1.1 (0.4–2.9)	0.34
Target vessel revascularization	2/55 (3.6%)	1/53 (1.9%)	1.89 (0.17–20.62)	8/360 (2.2%)	11/364 (3.0%)	0.73 (0.29–1.81)	1.0 (0.2–4.2)	0.38
Target lesion revascularization	3/55 (5.5%)	0/53 (0.0%)	-	6/360 (1.7%)	12/364 (3.3%)	0.50 (0.19–1.33)	1.2 (0.3–4.5)	< 0.01
Stent thrombosis	2/55 (3.6%)	1/53 (1.9%)	1.94 (0.18–20.85)	3/360 (0.8%)	4/364 (1.1%)	0.76 (0.17–3.38)	4.0 (1.0–15.9)	0.52
Acute renal insufficiency or renal replacement therapy	6/55 (10.9%)	10/53 (18.9%)	0.61 (0.22–1.72)	9/360 (2.5%)	3/364 (0.8%)	3.05 (0.83–11.26)	5.0 (2.3–10.7)	0.09
Major bleeding (BARC type 3 or 5)	5/55 (9.1%)	3/53 (5.7%)	1.63 (0.40–6.69)	8/360 (2.2%)	17/364 (4.7%)	0.47 (0.20–1.08)	2.0 (0.7–5.2)	0.14
Procedural success^**2**^	43/49 (87.8%)	35/40 (87.5%)	1.02 (0.29–3.64)	302/332 (91.0%)	270/295 (91.5%)	0.93 (0.53–1.62)		
Quality of life								
EQ-5D-5L index^**3**^	0.823 ± 0.326, *N* = 50	0.842 ± 0.305, *N* = 51	1.09 (0.71–1.69)	0.929 ± 0.176, *N* = 349	0.921 ± 0.180, *N* = 349	1.06 (0.90–1.23)		

*BARC*, bleeding academic research consortium; *EQ-5D-5L*, European Quality of life-5 dimension-5 level; *CI*, confidence interval; *HR*, hazard ratio

^*^Treatment effects are described by hazard ratios or odds ratios and their 95% confidence intervals

^1^Hazard ratio and 95% confidence interval, Fine and Gray method, considering competing risk all-cause death

^2^Odds ratio and 95% confidence interval, Wald method

^3^Wilcoxon–Mann–Whitney odds ratio and 95% confidence interval

^†^Adjusted HRs with corresponding CIs for the respective endpoints in patients with and without renal dysfunction. Adjustments were performed using a Cox-proportional hazards model with step-wise variable selection

**Fig. 3:**
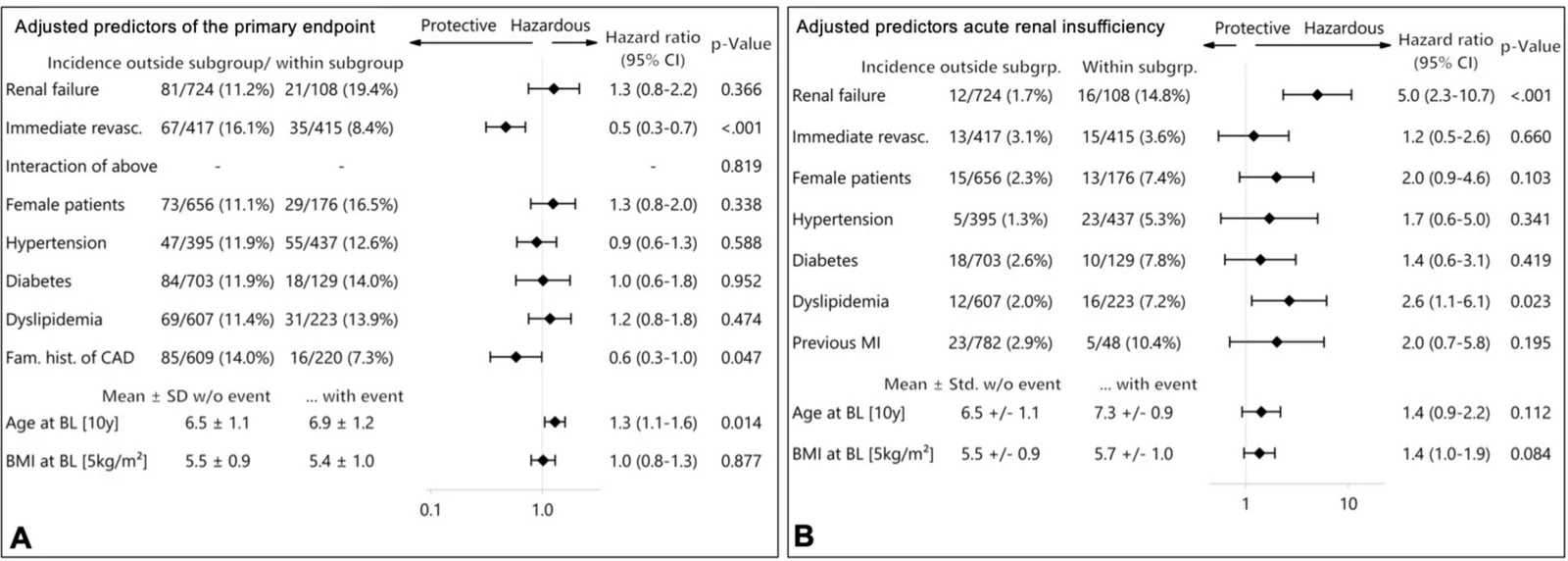
Multivariable predictors of the primary end point and renal end point. (**A** Death, non-fatal myocardial infarction, stroke, unplanned ischemia-driven revascularization, or hospitalization for heart failure. **B** Acute renal insufficiency or renal replacement therapy)

**Fig. 4:**
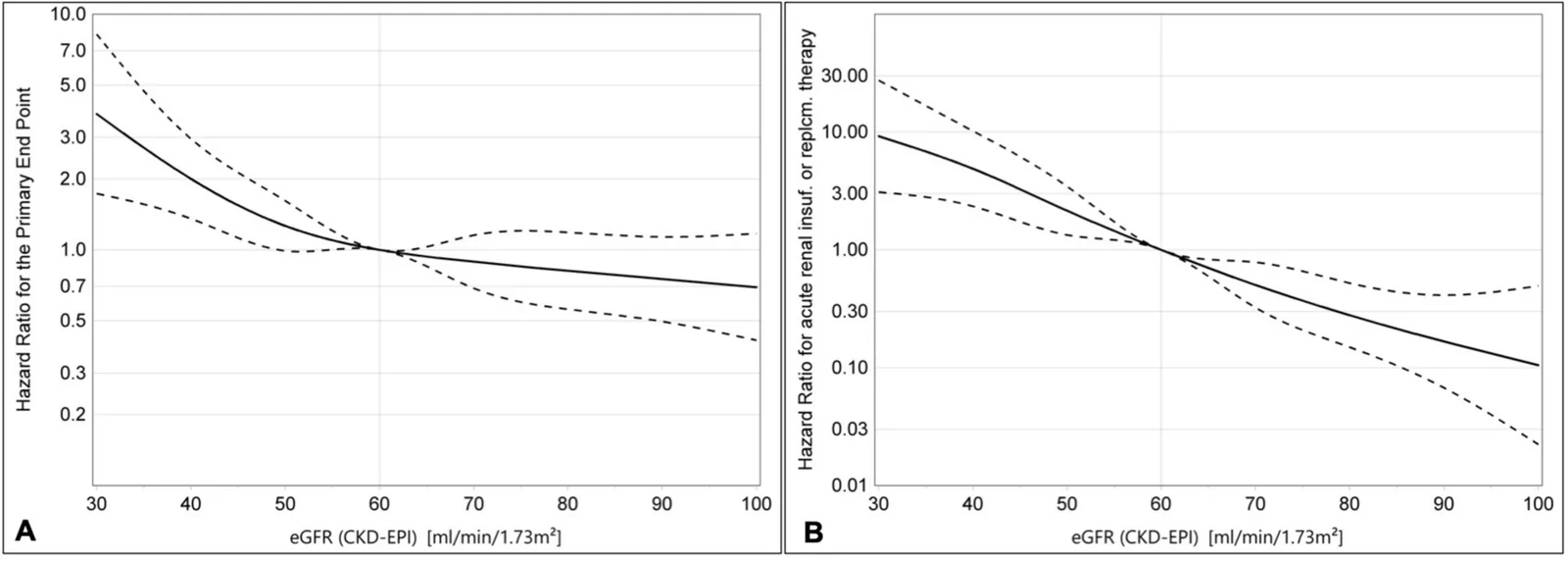
The continuous representation of eGFR and its association with the primary endpoint (**A**) and acute renal insufficiency or renal replacement therapy (**B**) using natural cubic splines. An eGFR of 60 ml/min/1.72 m^2^ was chosen as reference point for hazard ratios, resulting in a convergence of the HR-curve at 1.0. CKD-EPI, chronic kidney disease–epidemiology collaboration; eGFR, estimated glomerular filtration rate

After multivariable adjustments, age (adjusted HR per 10 year increment 1.3, 95% CI 1.1–1.6, *p* = 0.01), immediate multivessel PCI (adjusted HR 0.50, 95% CI 0.30–0.70, *p* < 0.01), and family history of coronary artery disease (adjusted HR 0.60, 95% CI 0.30–1.00, *p* = 0.05) were associated with the primary end point, but not renal dysfunction at baseline (adjusted HR 1.30, 95% CI 0.8–2.20, *p* = 0.36, Fig. 3). Thus, there was no significant interaction between renal dysfunction at baseline and the randomized treatment assignment with respect to the primary end point (*p* for interaction 0.82). The continuous representation of eGFR and its association with the primary endpoint using natural cubic splines are shown in Fig. 4. The randomized study group was still a significant predictor (*p* < 0.01) when the eGFR effect was included in the model as a continuous spline (*p* < 0.01). Interactions between study groups and eGFR were not apparent.

### Secondary end points in patients undergoing immediate or staged multivessel PCI

In patients with renal dysfunction at baseline undergoing immediate or staged multivessel PCI, unadjusted main effects for individual secondary end points were similar between groups. However, interpretability was limited, given the low absolute number of events (Table 3).

Rates of acute renal insufficiency or renal replacement therapy were numerically lower in patients with renal dysfunction at baseline who underwent immediate multivessel PCI (10.9% vs. 18.9%, unadjusted HR 0.61, 95% CI 0.22–1.72). After adjustment, renal dysfunction at baseline emerged as the strongest predictor for acute renal insufficiency or renal replacement therapy (adjusted HR 5.0, 95% CI 2.30–10.70, *p* < 0.01, Fig. 3). The continuous representation of eGFR and its association with acute renal insufficiency or renal replacement therapy using natural cubic splines are shown in Fig. 4 and was consistent with the dichotomized analysis. Adjusted hazard ratios for all other secondary end points are presented in Table 3, and full multivariable models for all-cause death, cardiac death, non-cardiovascular death, and major bleeding are given in the Supplementary Appendix (Supplementary Figures S1 – S4). After adjustment for confounders, renal dysfunction at baseline had no significant effect on these events.

## Discussion

The main findings of this pre-specified subgroup analysis of the MULTISTARS AMI trial are as follows:

- In patients presenting with STEMI and MVD, renal dysfunction at baseline was associated with an excess risk of all-cause death, non-fatal MI, stroke, unplanned ischemia-driven revascularization, or hospitalization for heart failure at 1 year.
- In patients with renal dysfunction at baseline, increased rates of major bleeding and acute renal failure were observed.
- The incidence of the primary end point and acute renal insufficiency or renal replacement therapy in patients undergoing an immediate compared to a staged multivessel PCI strategy did not appear to be significantly altered by the presence of renal dysfunction at baseline.
- When accounting for confounders, renal dysfunction at baseline emerged as strongest risk factor for the development of acute renal insufficiency or the need for renal replacement therapy.

Both the underlying acute condition (STEMI in patients with MVD), as well as the treatment with PCI and amount of contrast agent used might cause or worsen renal dysfunction [3]. In addition, renal function is often unknown at the time of primary PCI. Traditional measures to reduce the risk of AKI associated with PCI, such as pre- and post-procedural hydration, left ventricular end-diastolic pressure-guided hydration, the use of a limited amount of contrast agent according to the concept of the “maximal acceptable contrast dose” (MACD), and correction of modifiable risk factors (periprocedural correction of hypovolemia, hypotension, hyperglycemia, anemia, and nephrotoxic drugs) are often not feasible [6–8, 14–17]. As recently shown, hemodynamic support in patients presenting with STEMI and cardiogenic shock by the use of a microaxial flow-pump was associated with increased rates of renal replacement therapy, while lowering mortality [18].

Evidence to guide the decision on the optimal timing of non-culprit lesion PCI in consideration of renal function among patients with acute myocardial infarction and MVD is limited. The 2023 European Society of Cardiology Guidelines on the Management of Patients with Acute Coronary Syndromes (ACS) recommended to apply the same diagnostic and therapeutic strategies in patients with CKD as in patients with normal kidney function (class I, level of evidence C), and state that hydration during and after angiography should be considered in patients at risk of CIN, especially in patients with AKI and/or CKD with an eGFR < 30 mL/min/1.73 m^2^ (class IIa, level of evidence B) [9]. In the Complete versus Culprit-Only Revascularization Strategies to Treat Multivessel Disease after Early PCI for STEMI (COMPLETE) trial, comparing complete versus culprit lesion-only revascularization in patients with STEMI and MVD, 2% of patients had a history of CKD. Numerically more patients in the complete (with non-culprit lesion PCI being performed during the index hospitalization in 63% of patients) as compared to the culprit lesion-only revascularization group developed CIN (30 versus 19 patients, OR 1.59, 95% CI 0.89–2.84, *p* = 0.11). Only a quarter of these events were attributed to non-culprit lesion interventions [19].

In this subgroup analysis of the MULTISTARS AMI trial, renal dysfunction at baseline was associated with an excess risk of all-cause death, non-fatal MI, stroke, unplanned ischemia-driven revascularization, or hospitalization for heart failure at 1 year. Interestingly, this association was not observed in the percutaneous complete revascularization strategies using sirolimus-eluting biodegradable polymer-coated stents in patients presenting with acute coronary syndrome and multivessel disease (BIOVASC) trial which investigated the time point of non-culprit lesion PCI in patients with acute myocardial infarction (including both STEMI and non-ST segment elevation myocardial infarction [NSTEMI]) and MVD. Among the 764 randomized patients of the BIOVASC trial, neither CKD nor amount of contrast used was associated with the incidence of the primary end point, consisting of all-cause death, myocardial infarction, unplanned ischemia-driven revascularization, or cerebrovascular events at 1 year [20]. Differences in trial designs, baseline characteristics, and the inclusion of a broad NSTEMI patient population in the BIOVASC trial may at least in part explain these differences.

Irrespective of the presence/absence of renal dysfunction at baseline, an immediate multivessel PCI strategy appeared non-inferior to a staged multivessel PCI strategy with respect to the primary end point. The increased amount of contrast agent used during the index procedure did not seem to have affected outcomes when immediate multivessel PCI was performed.

Renal dysfunction at baseline was identified as strongest risk factor for the development of acute renal insufficiency or the need for renal replacement therapy. A retrospective analysis of a large PCI registry showed that although the development of acute renal failure was relatively infrequent (3.3%), a seven- to 13-fold increased incidence was observed in patients with pre-existing moderate to severe CKD [7]. Data on the impact of the amount of contrast agent used and the risk of future acute renal failure are controversial. In a retrospective analysis, each 100 ml of contrast agent used was associated with a 12% increased risk of acute renal failure, and patients who developed acute renal failure had a substantially higher risk of in-hospital as well as long-term risk of death, vascular complications, and bleeding [7]. Otherwise, in patients with multiple exposures to contrast media, established risk factors for CKD progression, but not the cumulative contrast volume, were associated with worsening of renal function over time [8]. Recently, a large analysis from the National Cardiovascular Data Registry (NCDR) including 455,806 PCI procedures provided contemporary estimates of AKI risk after PCI with an updated risk model. The strongest four predictors of AKI were salvage procedures, shock, unresponsive cardiac arrest, and pre-existing CKD with an eGFR < 30 ml/min/1.73 cm^2^. Among the wide range of selected variables, there were few that appear to be potentially modifiable (anemia, concomitant procedures, cardiovascular instability) [21]. Hence, avoidance of AKI and CIN after PCI seems important, given the strong association of AKI with adverse short- and long-term outcomes, including death. It seems reasonable to minimize the amount of contrast used and to focus on optimal fluid management in patients with STEMI and MVD, particularly in those at high risk for AKI, i.e., in those with pre-existing renal impairment. However, there is insufficient evidence for a specific upfront strategy to avoid renal deterioration.

Some limitations merit consideration. This subgroup analysis was not powered to detect meaningful differences between immediate and staged multivessel PCI strategies within this subgroup. Therefore, these results have to be regarded as exploratory and hypothesis generating. The trial excluded patients with severe renal failure (eGFR < 30 mL/min) or renal replacement therapy; thus, the results only apply to a subset of patients with moderate renal dysfunction, and results cannot be extrapolated to patients with more severe renal dysfunction at baseline. To which degree renal function was pre-existing, new, or worsened as a result from the acute presentation could not be investigated.

## Conclusions

In this pre-specified subgroup analysis of the MULTISTARS AMI trial, patients with renal dysfunction at baseline (within the eGFR range of 30 to 60 ml/min/1.73 m^2^) were at higher risk for adverse clinical outcomes, including the primary end point of cardiovascular events, major bleeding, acute renal insufficiency, and death. Renal dysfunction was the strongest predictor for subsequent acute renal insufficiency. The incidence of cardiovascular events and acute renal insufficiency or renal replacement therapy in STEMI patients undergoing immediate compared to staged multivessel PCI did not appear to be significantly altered by baseline renal dysfunction. These findings have to regarded as exploratory and hypothesis generating.

## Supplementary Information

Below is the link to the electronic supplementary material.

ESM 1 (PDF 867 KB)

## Data Availability

Data can be made available upon reasonable request to the corresponding author.
