# Supplementary material for: Impact of renal dysfunction on immediate versus staged revascularization of non-culprit lesions in patients with ST segment elevation myocardial infarction: a pre-specified subgroup analysis of the randomized MULTISTARS AMI trial
Source: Clin Res Cardiol. 2026 Mar 9;115(10):1831–44. doi: 10.1007/s00392-026-02875-w (PMC13680518; doi:10.1007/s00392-026-02875-w)
Supplement: Supplementary file 1 [file 392_2026_2875_MOESM1_ESM.pdf]

Supplementary Table 1: Medication at first discharge

Characteristics	Renal failure (eGFR<60)	No renal failure (eGFR≥60)	P-value
Aspirin	93/105 (88.6 %)	689/720 (95.7 %)	<.01
Clopidogrel	28/105 (26.7 %)	65/720 (9.0 %)	<.01
Prasugrel	37/105 (35.2 %)	341/720 (47.4 %)	0.02
Ticagrelor	39/105 (37.1 %)	310/720 (43.1 %)	0.25
Oral anticoagulation (VKA or NOAC)	19/105 (18.1 %)	44/720 (6.1 %)	<.01
Statin	101/105 (96.2 %)	704/719 (97.9 %)	0.27
ACEI or ATII antagonist	87/105 (82.9 %)	617/719 (85.8 %)	0.42
Beta blocker	91/105 (86.7 %)	612/719 (85.1 %)	0.68
Oral antidiabetics	28/105 (26.7 %)	94/720 (13.1 %)	<.01
Insulin	13/105 (12.4 %)	35/720 (4.9 %)	<.01

Values are n (%), or n/N (%).
ACE denotes angiotensin converting enzyme inhibitor, ATII, angiotensin II receptor, NOAC, non-vitamin K antagonist oral anticoagulant, VKA, vitamin K antagonist.

Supplementary Table 2: Procedural characteristics

Characteristics	Renal dysfucntion (eGFR<60)	No renal dysfunction (eGFR≥60)	P-value
Location of culprit lesion			
Left main coronary artery	1/108 (0.9 %)	0/724 (0.0 %)	<.01
Left anterior descending coronary artery	42/108 (38.9 %)	290/724 (40.1 %)	0.82
Left circumflex coronary artery	13/108 (12.0 %)	131/724 (18.1 %)	0.12
Right coronary artery	52/108 (48.1 %)	304/724 (42.0 %)	0.23
Location of non-culprit lesion undergoing PCI			
Left main coronary artery	0/94 (0.0 %)	5/664 (0.8 %)	0.40
Left anterior descending coronary artery	43/94 (45.7 %)	340/664 (51.2 %)	0.32
Left circumflex coronary artery	48/94 (51.1 %)	297/664 (44.7 %)	0.25
Right coronary artery	27/94 (28.7 %)	229/665 (34.4 %)	0.27
No. of vessels with relevant non-culprit lesions			
1	78/91 (85.7 %)	509/626 (81.3 %)	0.31
≥2	13/91 (14.3 %)	117/626 (18.7 %)	
Access site for index procedure			
Radial	72/108 (66.7 %)	533/724 (73.6 %)	0.13
Femoral	36/108 (33.3 %)	191/724 (26.4 %)	
Access site for staged procedure			
Radial	31/45 (68.9 %)	261/337 (77.4 %)	0.20
Femoral	14/45 (31.1 %)	76/337 (22.6 %)	
Method of PCI guidance			
Fractional flow reserve			
Index procedure	1.0/55.0 (1.8 %)	11.0/360.0 (3.1 %)	0.61
Staged procedure	2/45 (4.4 %)	34/337 (10.1 %)	0.22
Intravascular ultrasound			
Index procedure	0/55 (0.0 %)	8/360 (2.2 %)	0.26
Staged procedure	1/45 (2.2 %)	7/337 (2.1 %)	0.95
Optical coherence tomography			
Index procedure	0/55 (0.0 %)	2/360 (0.6 %)	0.58
Staged procedure	0/45 (0.0 %)	7/337 (2.1 %)	0.33
Stents used per patient			
Index procedure	2.0 (1.0, 3.0), N=108	2.0 (1.0, 3.0), N=722	0.70
Index plus staged procedure	3.0 (2.0, 4.0), N=97	3.0 (2.0, 4.0), N=671	0.24

Total stent length - mm			
Index procedure	44.0 (32.0, 68.0), N=108	46.0 (30.0, 70.0), N=722	0.96
Index plus staged procedure	64.0 (44.0, 92.0), N=97	68.0 (48.0, 96.0), N=671	0.56
Volume of contrast material - ml			
Index procedure	196.0 (144.0, 250.0), N=107	200.0 (150.0, 274.0), N=719	0.06
Index plus staged procedure	257.0 (191.0, 342.0), N=100	290.0 (220.0, 380.0), N=688	<.01
Duration of fluoroscopy – min			
Index procedure	14.0 (8.0, 22.0), N=107	14.0 (9.0, 20.0), N=711	0.99
Index plus staged procedure	20.0 (13.0, 28.0), N=100	20.0 (14.0, 30.0), N=676	0.61
Duration of procedure – min			
Index procedure	61.0 (47.5, 80.5), N=108	61.0 (48.0, 81.0), N=721	0.88
Index plus staged procedure	85.0 (66.0, 106.0), N=100	86.0 (65.0, 120.0), N=689	0.47
Time from index to staged procedure - days	33.0 (25.0, 40.0), N=45	38.0 (30.0, 43.0), N=337	0.02
Hospital stay – days			
Index procedure	5.0 (4.0, 7.0), N=103	4.0 (3.0, 6.0), N=709	<.01
Index plus staged procedure	5.0 (4.0, 7.0), N=98	5.0 (4.0, 6.0), N=677	0.01

Values are median (Q1-Q3), n (%), or n/N (%).
PCI, percutaneous coronary intervention.

Supplementary Figure S1: Multivariable analysis for all-cause death at 1 year

Adjusted Predictors of All-Cause Death

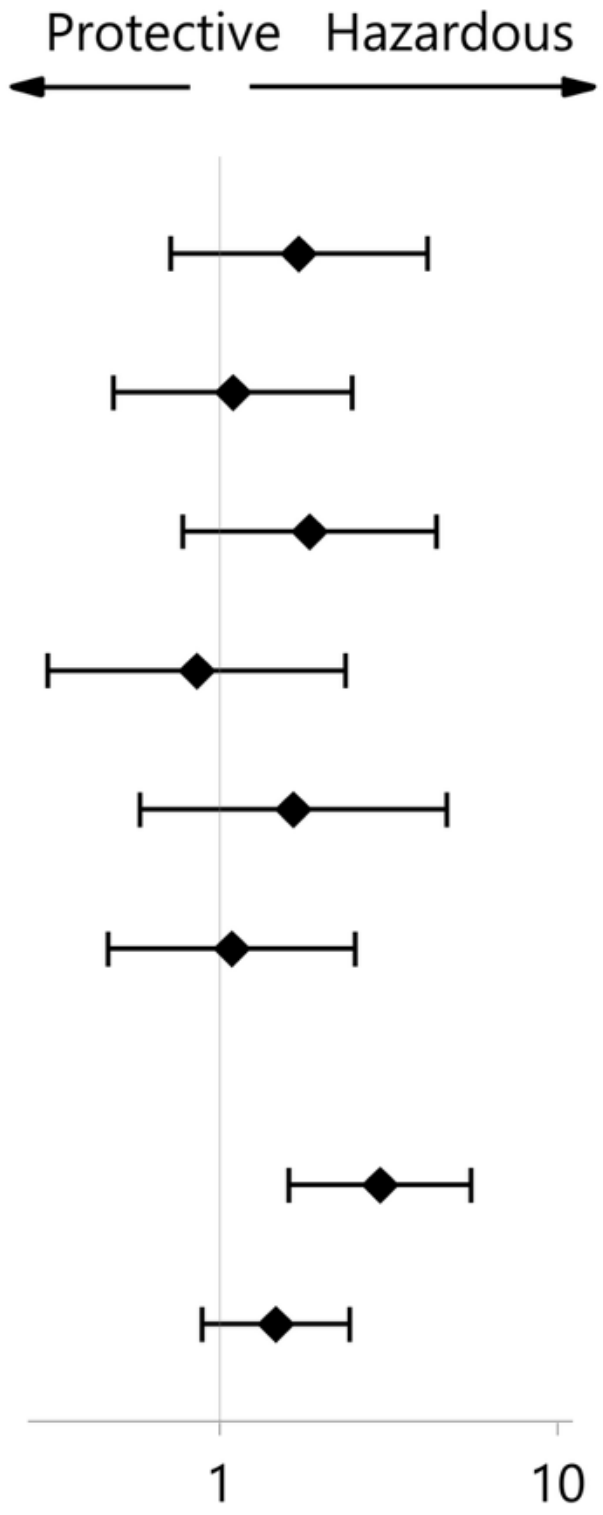

	Incidence outside subgrp.	Within subgrp.	Hazard ratio (95% CI)	p-Value
Renal failure	13/724 (1.8%)	9/108 (8.3%)	1.7 (0.7-4.1)	0.225
Immediate revasc.	10/417 (2.4%)	12/415 (2.9%)	1.1 (0.5-2.5)	0.824
Female patients	11/656 (1.7%)	11/176 (6.3%)	1.9 (0.8-4.4)	0.162
Hypertension	7/395 (1.8%)	15/437 (3.4%)	0.9 (0.3-2.4)	0.765
Diabetes	15/703 (2.1%)	7/129 (5.4%)	1.7 (0.6-4.7)	0.346
Dyslipidemia	15/607 (2.5%)	7/223 (3.1%)	1.1 (0.5-2.5)	0.848
	Mean ± Std. w/o event	… with event		
Age at BL [10y]	6.5 +/- 1.1	7.7 +/- 1.0	3.0 (1.6-5.6)	<.001
BMI at BL [5kg/m^2]	5.5 +/- 0.9	5.6 +/- 1.2	1.5 (0.9-2.4)	0.134

Legend: BL: baseline; BMI: body mass index; CI: confidence interval; Std: standard deviation.

Supplementary Figure S2: Multivariable analysis for cardiac death at 1 year

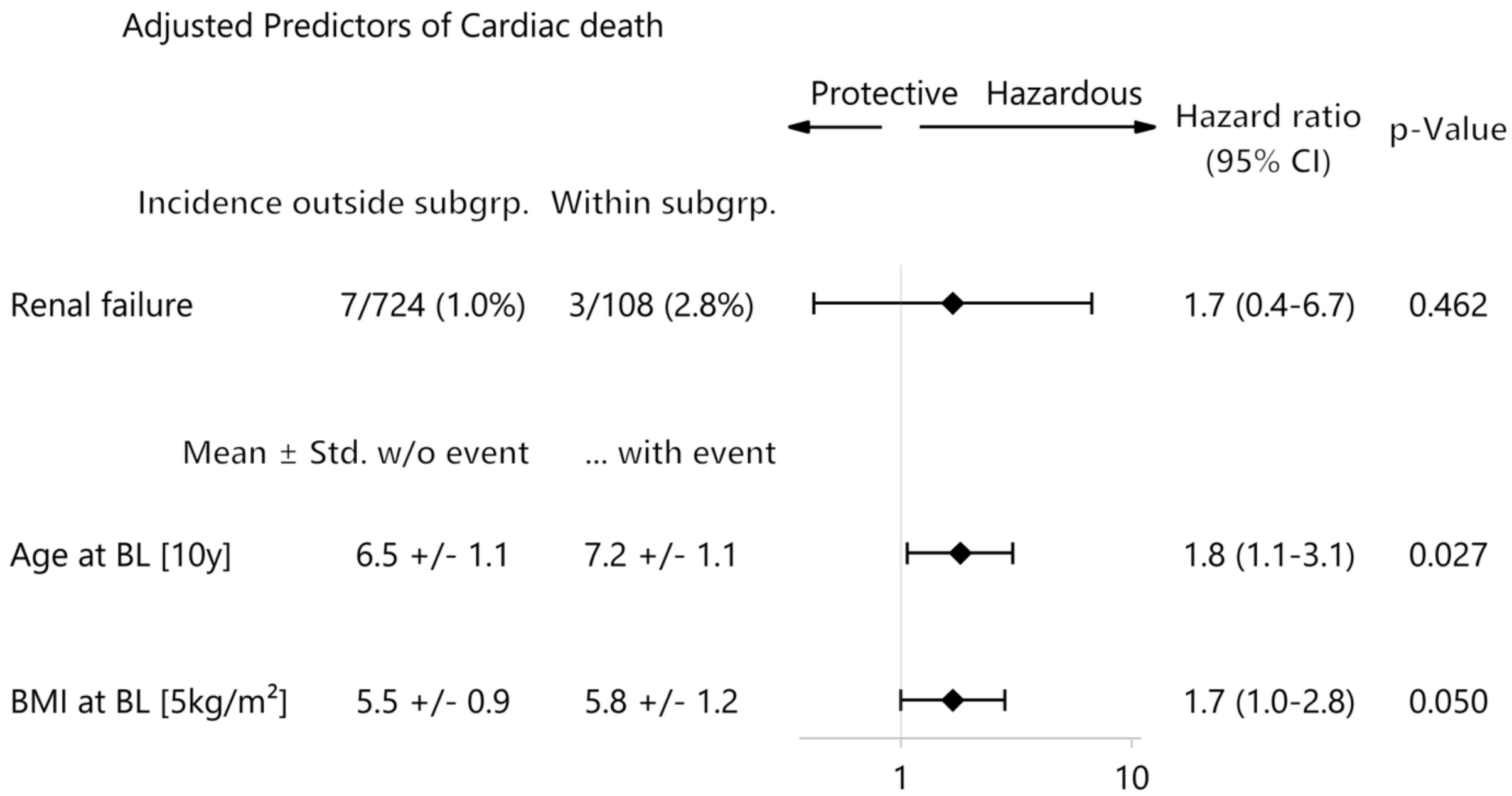

Legend: BL: baseline; BMI: body mass index; CI: confidence interval; Std: standard deviation.

Supplementary Figure S3: Multivariable analysis for non-cardiovascular death at 1 year

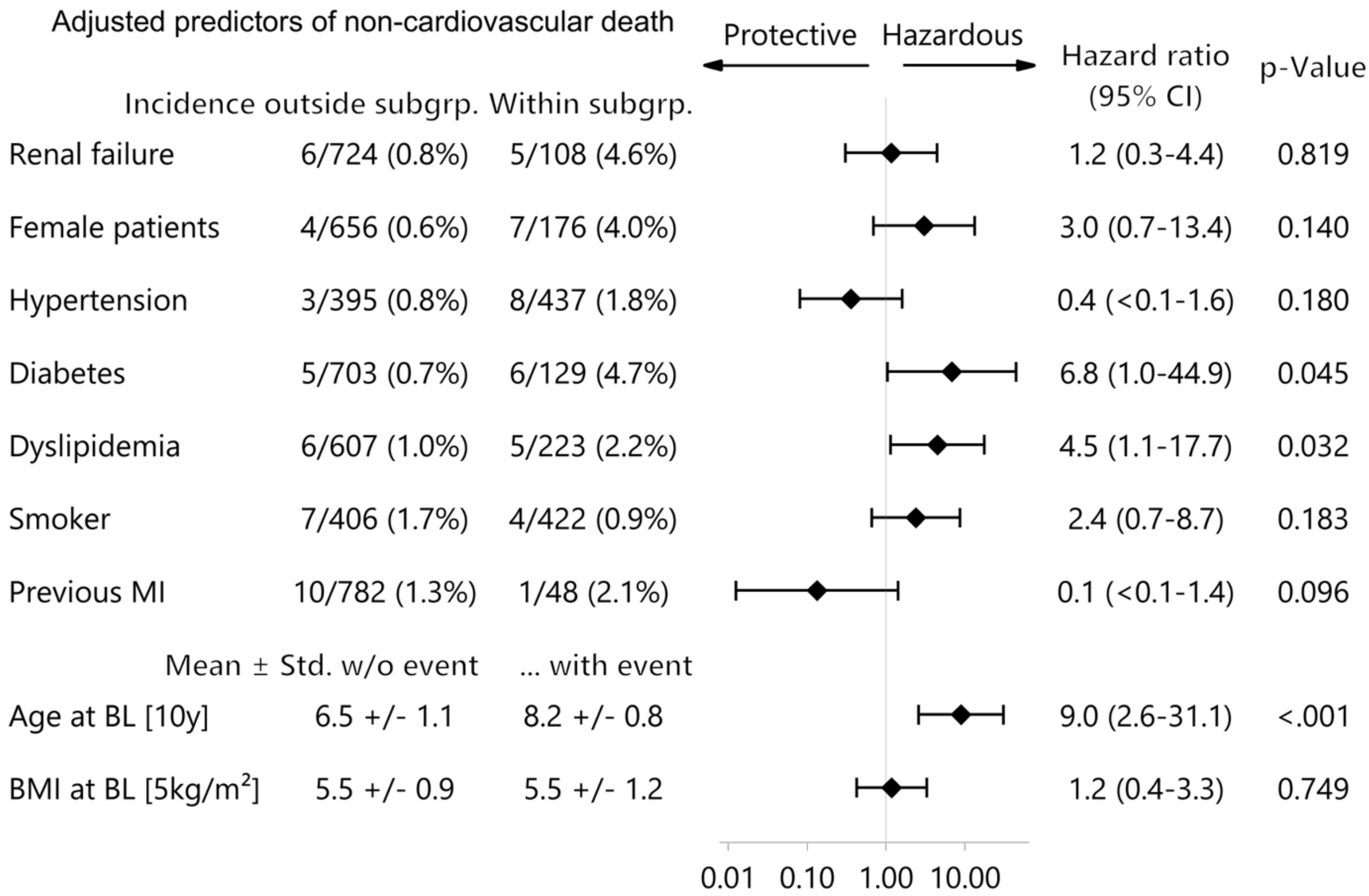

Legend: BL: baseline; BMI: body mass index; CI: confidence interval; MI: myocardial infarction; Std: standard deviation.

Supplementary Figure S4: Multivariable analysis for major bleeding at 1 year

Adjusted Predictors of major bleeding

	Incidence outside subgrp.	Within subgrp.	Hazard ratio (95% CI)	p-Value
Renal failure	25/724 (3.5%)	8/108 (7.4%)	2.0 (0.7-5.2)	0.178
Immediate revasc.	20/417 (4.8%)	13/415 (3.1%)	0.6 (0.3-1.3)	0.193
Female patients	27/656 (4.1%)	6/176 (3.4%)	0.7 (0.3-1.8)	0.472
Hypertension	14/395 (3.5%)	19/437 (4.3%)	0.9 (0.4-2.1)	0.858
Diabetes	25/703 (3.6%)	8/129 (6.2%)	1.3 (0.5-3.4)	0.531
Dyslipidemia	23/607 (3.8%)	9/223 (4.0%)	0.7 (0.3-1.8)	0.479
Previous PCI	28/775 (3.6%)	5/56 (8.9%)	2.7 (0.9-8.2)	0.080
	Mean ± Std. w/o event	... with event		
Age at BL [10y]	6.5 +/- 1.1	6.7 +/- 1.2	1.2 (0.8-1.7)	0.374
BMI at BL [5kg/m^2]	5.5 +/- 0.9	5.6 +/- 1.1	1.2 (0.8-1.8)	0.396

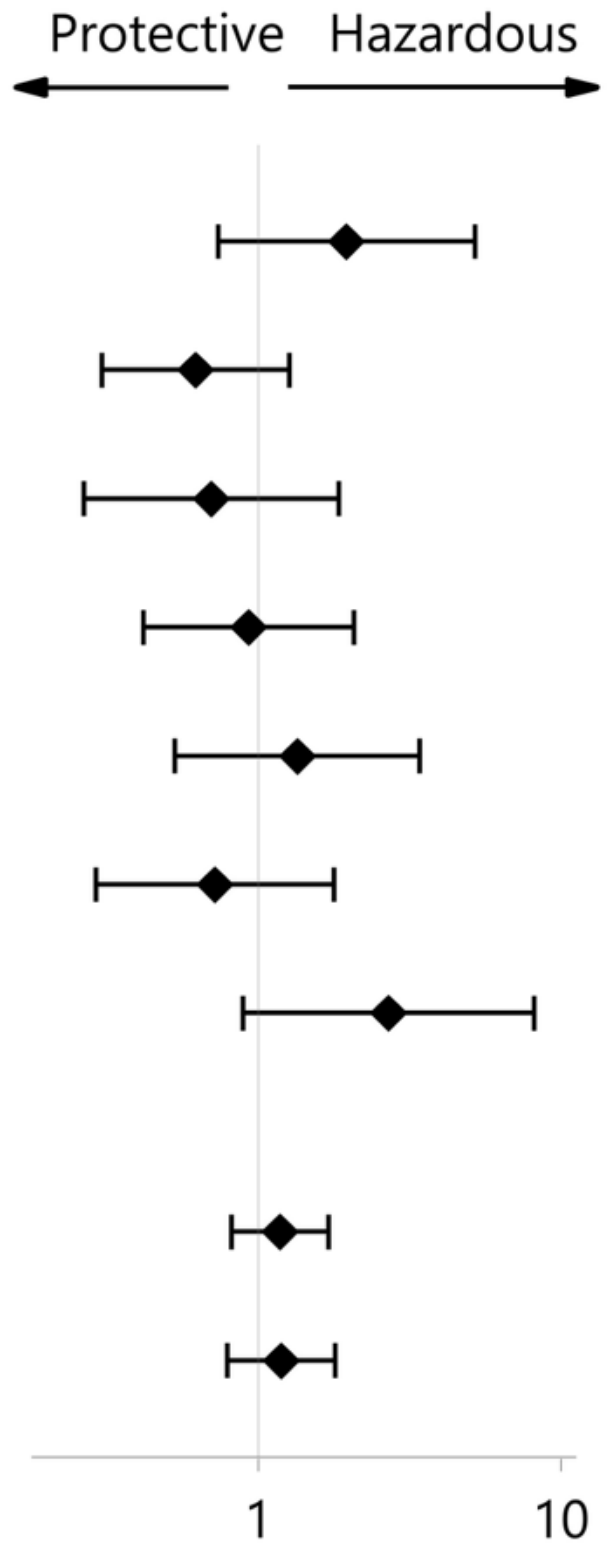

Major bleeding: defined as Bleeding Academic Research Consortium grade 3 or 5
Legend: BL: baseline; BMI: body mass index; CI: confidence interval; PCI: percutaneous coronary intervention; Std: standard deviation.
